# Assessment of the Functional Quality of Extra Virgin Olive Oil: Green Extraction of Phenolic Compounds Using Ethyl Lactate

**DOI:** 10.3390/foods14223822

**Published:** 2025-11-07

**Authors:** Chrysostomos Tsitsipas, Athanasios Gerasopoulos, Nikolaos Nenadis, Dimitrios Gerasopoulos

**Affiliations:** 1 Laboratory of Food Processing and Engineering, Department of Food Science and Technology, Faculty of Agriculture, Aristotle University of Thessaloniki, 54124 Thessaloniki, Greece; ctsitsip@agro.auth.gr; 2 Laboratory of Food Chemistry and Technology, School of Chemistry, Aristotle University of Thessaloniki, 54124 Thessaloniki, Greece; ageraso@chem.auth.gr (A.G.); niknen@chem.auth.gr (N.N.)

**Keywords:** phenolic compounds, extraction solvents, hydroxytyrosol, tyrosol, DPPH, antioxidant

## Abstract

Phenolic compounds are regarded as one of the components responsible for olive oil’s functional properties and health benefits. These chemicals act as antioxidants and anti-inflammatories, and prevent chronic diseases. The Folin–Ciocalteu reagent or HPLC procedures are commonly used to determine the concentration of total phenolic compounds in olive oil. The use of ethyl lactate or lactic acid ethyl ester (LAEE) instead of methanol (MeOH) was examined in terms of green chemistry. Six extra virgin olive oils (EVOOs) with phenolic content ranging from 20 to 350 mg/L, were first extracted with 1:4, 2:3, 3:2, 4:1, and 5:0 MeOH or LAEE/water, (*v*/*v*), to determine total phenolic content (TPC) and antiradical activity (%RSA) using the Folin–Ciocalteu reagent and DPPH assay, respectively. The concentration of extracted phenolics or extracts’ RSA increased as the water content in the organic solvent mixture decreased. Also, TPC values were greater when extracted with LAEE than MeOH, while the differences were modest. The HPLC profiles of EVOO phenolic extracts produced by 4:1 MeOH or LAEE/water, (*v*/*v*), were indistinguishable in principal component analysis. Simplification of the phenolic profile via acid hydrolysis, resulting in increased hydroxytyrosol and tyrosol content liberated from the corresponding bound forms, showed that both organic solvents equally recovered the predominating phenols of the polar fraction. A noted limitation of LAEE extraction is the need for freeze-drying to remove it prior to HPLC analysis of aqueous extracts. Nonetheless, these findings support LAEE as an effective and environmentally friendly alternative to MeOH for EVOO phenolic extraction in both analytical and industrial contexts.

## 1. Introduction

Plant phenolics are commonly extracted using traditional organic solvents such as methanol and ethyl acetate, or organic solvents such as ethanol and water that are generally regarded as safe (GRAS), or a combination of the two [1]. Chemat et al. [2] proposed that “Green extraction is based on the discovery and development of extraction processes that reduce energy consumption, allow the use of alternative organic solvents and renewable natural products, and ensure a safe and high-quality extract or product”. Another GRAS organic solvent is ethyl lactate (Lactic acid ethyl ester-LAEE), a carbohydrate fermentation product that meets eight of the twelve green chemistry criteria [3]. Lactate esters are readily biodegradable, suggesting little concern from an environmental point of view. Furthermore, the information available on lactate esters suggests that these materials should not present any potential health risk during use [4]. The European Food Safety Authority [5] and the United States Food and Drug Administration (FDA) [6] have allowed its usage in the manufacture of food.

LAEE has gained popularity as an extraction and separation agent in place of traditional organic solvents over the last decade. This suggests that this organic solvent may have applications in natural product chemistry; relevant reports include the extraction of sclareol [7], quercetin and rutin [8], caffeine [9], amino acids [10], and phenols [11] from plant material. LAEE has also been shown to extract carotenoids [12], as well as tocopherols [13].

Olive oil, a popular staple of the “Mediterranean diet,” is known for its high concentration of phenolic compounds, among other components. Olive oil phenols are one of the most widely explored categories of natural food antioxidants, with an increasing emphasis on their several bioactivities that promote human health [14]. It has been demonstrated that phenolic compounds’ antiradical scavenging properties are beneficial to human health [15,16,17,18].

Sample preparation is widely recognized as one of the bottlenecks inherent in analytical techniques because it affects both performance and the greenness of the analysis. Sample preparation receives the most attention in the analytical process because it is considered the most difficult to carry out. Sample preparation of olive oil phenolics often entails extraction techniques, which are presently being investigated in terms of ease of implementation, time consumption, efficiency, cost, and, most recently, greenness.

In the past, olive oil phenol analysis attracted researchers’ attention [19,20,21,22]. The most common liquid–liquid extraction procedures use various organic solvents and conventional approaches of manual or mechanical agitation [21,23,24,25]. Currently, the official method of analysis for phenolics approved by the IOOC [26] proposes the use of 80% aqueous methanol (MeOH) (or 4:1 MeOH-to-water ratio, *v:v*) and sonication to prepare samples of phenolic compounds extracted from olive oil [27]. Following extraction, total olive oil phenols can be determined using the Folin–Ciocalteu reagent (which is not specific for phenols) followed by analysis by UV and analysis by HPLC (which is limited by the extraction procedure and the complexity of the phenolic fraction).

Emerging green extraction methods for polyphenols from edible oils include green solvents like ionic liquids, and bio-based solvents, including deep eutectic solvents (DESs) [28] often combined with advanced techniques such as supercritical fluid extraction, ultrasound-assisted extraction, and microwave-assisted extraction [29]. These approaches reduce solvent use, energy consumption, and processing time while increasing extraction yields and maintaining the integrity of polyphenols.

LAEE is considered to be a potentially useful organic solvent for plant phenolic extraction since it is miscible with both hydrophilic and hydrophobic molecules such as polyphenols [30]. Furthermore, as a proton donor or acceptor, the hydrogen bonding reported in other lactate alpha-hydroxyesters [31] allows for intra- and intermolecular interaction [32]. However, because LAEE dissolves in paraffin oils, van der Waals interactions are also included [33]. As a result, this LAEE has a diversity of organic solvent characteristics that can be used in the extraction of a wide range of solutes.

In the context of green chemistry, the extraction qualities of LAEE were exploited for the isolation of metabolites from *Ambrosia arborescens*; it was reported that microwave-assisted extraction associated with LAEE could take the place of the traditional methanol maceration [34]. Using MeOH or LAEE produced similar phenolic profiles. According to [1], LAEE has been found to be an effective organic solvent for extracting polyphenols from *Cytisus scoparius*, producing extracts with high concentrations of plant phenolics including flavonoids, flavones, and the non-flavonoid phenolic compounds caffeic and protocatechuic acids and 3,4-dihydroxybenzaldehyde, as well as antioxidant activity. Furthermore, phenolic compounds were successfully extracted in an aqueous two-phase systems congaing LAEE, potassium sodium tartrate, or disodium succinate and water at 298.2 K and 0.1 MPa rutin [8].

Since olive phenols comprise a diverse spectrum of phenol constituents with high structural diversity and physicochemical behavior, a complete recovery from the matrix presents a difficult task [14]. However, to our knowledge, there are virtually no reports on phenolic extraction from olive oil although LAEE may appear promising in such extraction due to its properties.

This study compared the extraction of phenolic components from six extra virgin olive oils with varying phenolic concentrations using LAEE and MeOH. The effects of 1:4–5:0 LAEE- or MeOH-to-water ratios (*v:v*) were evaluated for total phenolic content and antioxidant activity; results on the comparative efficacy of the two organic solvents at the 4:1 organic solvent (LAEE or MeOH)-to-water ratio (*v:v*) were confirmed using HPLC-DAD phenolic profiling either before or after acid hydrolysis. As this study focuses on a comparative evaluation of two extraction solvents under identical analytical conditions; the same methodological approach was consistently applied to both sets of extracts.

## 2. Materials and Methods

### 2.1. Reagents and Materials

All reagents were of analytical grade. Lactic Acid Ethyl Ester (>99.0%) was obtained from Fluka (Buchs, Switzerland). The HPLC mobile phase was prepared using water, methanol, and acetonitrile HPLC-grade from Chem-Lab (Zedelgen, Belgium). Folin–Ciocalteu’s phenol reagent, sodium carbonate, glacial acetic acid, syringic acid, DPPH (2,2-diphenyl-picrylhydrazyl), caffeic acid, and HPLC standards (hydroxytyrosol, tyrosol, vanillic acid, vanillin, p-coumaric acid, ferulic acid, oleuropein, oleacin, oleocanthal, cinnamic acid, apigenin, syringic acid) were all purchased from Sigma-Aldrich, (St. Louis, MO, USA). A Kern 770 balance (Balingen, Germany) was used to prepare all liquid solutions gravimetrically, with a precision of ±0.0001 g.

### 2.2. Olive Oil Samples

This study used six Greek olive oil samples, labeled A, B, C, D, E, and F. The oil samples were collected during the 2023–2024 (A, B, C, and F) and 2024–2025 (D and E) harvest seasons in Crete and Central Macedonia, Greece. They all met the European Regulation’s standards for extra virgin olive oils (EVOOs) [35].

### 2.3. Extraction

All EVOOs with varied phenolic concentrations were collected earlier and kept in a freezer (−18 °C). The samples were defrosted, homogenized, and extracted with 20, 40, 60, 80, and 100% of aqueous methanol (MeOH) or aqueous ethyl lactate (LAEE) corresponding to *v*/*v* ratios of 1:4, 2:3, 3:2, 4:11, and 5:0. The mixtures were prepared by vortexing for 30 s. According to the IOC [36], the extractant volume/mass ratio for EVOO samples was 6 mL:2 g (*v:w*); also an internal standard (syringic acid, 1 mL, 0.015 mg/mL, in MeOH or LAEE, respectively) was added to the EVOOs, before the extraction. The organic solvent–EVOO mixture was vortexed for 1 min and sonicated for 15 min at 25 °C, at maximum power with Bandelin Sonorex Digiplus water bath (Berlin, Germany). Following this, the mixtures were centrifuged for 15 min at 5500 g (Hettich Universal, Tuttlingen, Germany), and the MeOH or LAEE phases were collected. These were further used to determine the total phenolic compound and antiradical scavenging activity. They were also employed to analyze the phenolic profile via HPLC. This procedure was carried out in triplicate, for each EVOO sample.

### 2.4. Determination of Total Phenolic Content

The total phenol content was determined colorimetrically, using the Folin–Ciocalteu reagent as described by Singleton et al. [37] and Scalbert et al. [38] with some modifications. LAEE or MeOH were used as controls, and 1 mL was diluted with 3 mL deionized water, then mixed with 0.25 mL Folin–Ciocalteu reagent, after 1 min with 0.75 mL of Na_2_CO_3_ (20% *w*/*v*), and kept in a dark environment at room temperature (at 20 °C) for 60 min. The absorbance of the solution was monitored at 760 nm using a Genesys 180 UV-VIS (Thermo Fisher Scientific, Waltham, MA, USA) spectrophotometer. For the blank, 1 mL of aqueous methanol or ethyl lactate was added. Total phenolic compounds (TPCs) were determined from the linear regression equation of standard curve (y = 0.0301x + 0.0261, R^2^ = 0.990) and expressed as caffeic acid equivalents (CAE g/L). When necessary, the samples were diluted by a suitable mixture of MeOH or LAEE; H_2_O and the calibration curve was established from 0 to 100 mg/L. All analyses of EVOO extracts were carried out in duplicate (n = 6), and handling of the reagents was performed in conditions as dark and cold as possible.

### 2.5. Determination of Radical Scavenging Activity

The antiradical properties of samples were determined using DPPH as a free radical according to Brand-Williams et al. [39] and Nenadis and Tsimidou [40] with some modifications. LAEE or MeOH on its own was used as control (0.1 mL), which was added to 3.9 mL of DPPH methanolic solution (100 μΜ) in a test tube. The tubes were then vortexed and kept in a dark environment at room temperature for 30 min (at 20 °C). The absorbance of the solution was monitored at 517 nm using a Genesys 180 UV-VIS (Thermo Fisher Scientific, Waltham, MA, USA) spectrophotometer.

The antioxidant activity of the olive oil extracts, expressed as (%) values (%RSA), were determined by using the following formula (after correction with appropriate blanks):

(1) % RSA = [(Abs517(t = 0) − Abs517(t)) × 100]/Abs517(t = 0)

where Abs refers to the absorbance of a blank sample (t = 0) and to the absorbance of an analyzed sample (t). All measurements were carried out in duplicate (n = 6).

### 2.6. HPLC Phenolic Profile Analysis

The qualitative and quantitative analysis of TPC in each MeOH and LAEE extract were carried out using high-performance liquid chromatography (syringic acid was added to EVOO as an internal standard). A 2 mL aliquot of the MeOH extract (containing syringic acid) was filtered using a 0.45 μm PVDF filter.

LAEE extracts were frozen first at −20 °C for 24 h, then freeze-dried (Sigma Christ 1–2 LD plus, Osterode am Harz, Germany) for 60 h until completely dry according to preliminary experiments (longer times may result in partial loss of low molecular weight analytes). LAEE, though of low volatility (boiling point of 154 °C), can be efficiently removed under high vacuum during freeze-drying, as also demonstrated in the literature involving other green solvents such as NADES [41].

Then, reconstitution was performed with 80% aqueous MeOH (2 mL) and 1 mL hexane, followed by vortexing and centrifugation at 6000× *g*. After phase separation, the hexane phase was removed, and the aqueous MeOH phase was filtered through a 0.45 mm filter before being analyzed directly using HPLC-DAD.

The phenolic compounds were separated using an HPLC-DAD system (ECOM, Prague, Czech Republic) with a Brisa “LC2” C18 column (5.0 μm, 150 × 4.6 mm, Teknokroma, Barcelona, Spain) at 25 °C. The elution was performed in gradient mode with a three-phase organic solvent mixture consisting of water acidified with 0.2% acetic acid (solvent A), methanol (solvent B), and acetonitrile (solvent C). A linear gradient was run from 96% (A), 2% (B), and 2% (C) to 50% (A), 25% (B), and 25% (C) during 40 min; it changed to 40% (A), 30% (B), and 30% (C) for 5 min; and then it changed to 0% (A), 50% (B), and 50% (C) for 25 min, followed by 12 min re-equilibration to the initial solvent composition. The mobile phase flow rate was 1 mL/min, and each sample had an injection volume of 50 μL. All phenolic compounds were identified by comparing retention times to those of standards (hydroxytyrosol, tyrosol, vanillic acid, vanillin, p-coumaric acid, ferulic acid, oleuropein, oleacin, oleocanthal, cinnamic acid, apigenin, syringic acid). HPLC analyses of EVOO extracts were carried out in triplicate (n = 3).

### 2.7. Acid Hydrolysis

The experimental procedure was followed according to Mulinacci et al. [42], with some modifications. An aliquot (200 μL) of the 80% methanolic final extract (MeOH or LAEE) was combined with 200 μL of a 1 M H_2_SO_4_ solution. The mixture was vortexed for 10 s before being incubated at 80 °C for 2.5 h in a water bath (Sonorex Digiplus, Germany). The hydrolysates were diluted with 200 μL of 80:20 MeOH/water (*v*:*v*). The three replicates were mixed, filtered via a 0.45 μm pore size PTFE membrane, and then injected into the chromatograph. The phenolic profile of the samples was subsequently evaluated in triplicate using HPLC, as previously described (2.6). Syringic acid was employed as an internal standard to evaluate the technique. This procedure was carried out in triplicate (n = 3).

### 2.8. Statistical Analyses

The design of the statistical analysis was completely randomized; two dependent variables (TPC and DPPH) and three factors (EVOOs, organic-solvent-to-water ratio (OSWR and organic-solvent-type (MeOH or LAEE))) were used in a three-way ANOVA with three replications per treatment. The factors’ major effects and interactions were examined. The data mean was separated using Tukey’s honestly significant difference test. In addition, principal component analysis (PCA) was utilized to identify variable interactions. Statistical analyses were carried out using the SPSS statistical software for Windows (version 29).

## 3. Results and Discussion

### 3.1. Total Phenolic Compounds

The effect of the main factors: EVOOs, the ratio of organic solvent to water (OSWR) (*v*:*v*), the selected organic solvent (MeOH and LAEE), and their interactions on variables TPC and %RSA were assessed using the partial eta-squared (η^2^ₚ), and this is displayed in Supplementary Table S1. The EVOOs as well as OSWR used had a very large and significant impact (η^2^ₚ = 1.00) on variable TPC. The type of organic solvent utilized had a significant impact on TPC extraction (η^2^ₚ = 0.85), whereas the interaction of organic solvent type with OSWR caused moderate effects.

#### 3.1.1. Effects of EVOOs

**Figure 1:**
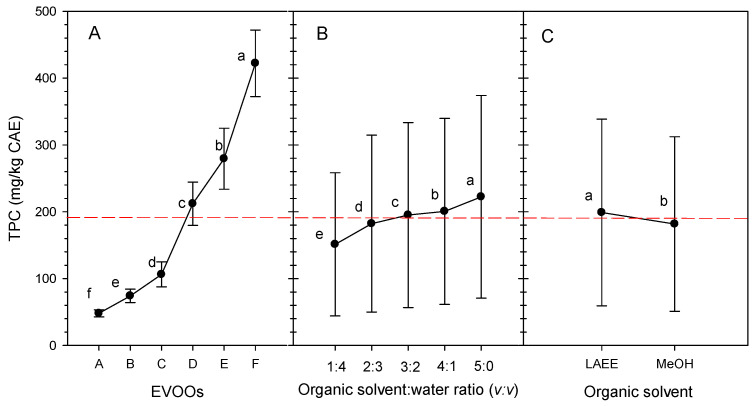
Main effects and means for the variable TPC (phenolic compounds, mg CAE/kg) obtained from the extraction of (**A**) six EVOOs using (**B**) 1:4 to 5:0 organic-solvent-to-water ratios (*v*:*v*), the organic solvents being (**C**) LAEE and MeOH. The dashed line represents mean value of all factors. Different letters at each point represent statistically significant differences according to Tukey’s test.

The average TPC for EVOOs was 195 mg/kg (Figure 1). Three EVOOs, namely A, B, and C, ranged from 40 to 105 mg/kg, while EVOOs D and E were in the middle range (215 and 280 mg/kg, respectively) and EVOO F peaked at 420 mg/kg CAE (Figure 1A).

TPC levels in EVOOs vary between 40 and 530 mg GAE/kg oil [43]. Other studies have found comparable contents, with olive oils ranging from 52 to 315 mg GAE/kg [44]. The average concentration of olive oil TPC in different reports [45,46,47,48,49,50] ranges from 100 to 300 mg/kg but can vary significantly depending on factors such as variety, geography, growing conditions, fruit maturity, and processing.

#### 3.1.2. Effects of Organic-Solvent-to-Water Ratio

EVOOs were extracted with organic solvents LAEE and MeOH at 1:4 to 5:0 OSWR (*v:v*) before TPC determination. EVOO phenolics have been reported to be extracted using different classical pure organic solvents (5:0, ratio of organic solvent to water, *v:v*) such as MeOH [27], ethanol [51,52], acetonitrile [53,54], and ethyl acetate [50], N-N Dimethylformamide [55], or even water [51]. Recent studies [56,57], have used natural deep eutectic systems to extract TPC from EVOOs.

In the case of MeOH, the reported OSWRs that have been utilized for the extraction of phenolics from EVOO are MeOH/water 4:1 (*v:v*) [27,50,58,59,60], 3:2 (*v:v*) [44,49,54,58,59,60,61,62], and 1:1 (*v:v*) [27,51,55].

The average TPC of EVOOs in this study varied greatly according to the OSWR used (Figure 1B). The lower the amount of water used to dilute the organic solvents, the greater the average concentration of extracted phenolics; an organic-solvent-to-water ratio of 3:2 yielded average extracted phenolics of 205 mg/kg, while a 4:1 ratio yielded 210 mg/kg. However, extraction with pure organic solvents (5:0, ratio of organic solvent to water, *v:v*) resulted in the highest average phenolic concentration in extracts (230 mg/kg). Jerman Klen and Vodopivec, ref. [27], similarly reported that pure MeOH yielded the highest recoveries, followed by MeOH/water mixtures of 4:1 and 1:1 (*v:v*).

On the other hand, utilizing a 4:1 MeOH/water mixture (*v*:*v*) for phenolic extraction of EVOO has been shown to extract higher phenolic concentrations than a 1:1 [25] or 3:2 ratio [54], although such an extract contains significant amounts of pigments and lipids.

#### 3.1.3. Effect of Organic Solvent

The average TPC value of EVOOs extracted with LAEE was 200 mg/kg, considerably different from those extracted with MeOH (>5%, *p* < 0.05), while the differences were small (Figure 1C). LAEE is classified as a polar-protic organic solvent since it can form hydrogen bonds while also exhibiting polar properties. This polarity (dielectric constant: 15.70 at 25 °C [32] enables LAEE to be completely miscible with water (dielectric constant: 78.5 at 25 °C) [63], as well as dissolve nonpolar hydrocarbons, making it a versatile, amphiphilic organic solvent [32,64]. MeOH is a well-known polar molecule (dielectric constant: 32.70 at 25 °C) [63] that is soluble in polar organic solvents (such as water) and can dissolve some polar organic compounds, such as phenols. Jessop [65] reported that the π* value (polarity and polarizability) of MeOH is 0.6 while that of LAEE is 0.8, when compared to water at 1. MeOH has a β value (basicity or hydrogen-bond-accepting ability) of 0.6, while LAEE has a value of 0.55, compared to water’s 0.15. These properties, including the aforementioned dielectric constant ones, qualifies LAEE as a polar organic solvent, and mixing LAEE with water improves its capacity to extract molecules with greater polarity. As previously noted, MeOH combined with water has been extensively employed as an organic solvent for extracting EVOO phenolics. However, LAEE has had little application thus far; it has been reported to be employed for the extraction of tocopherols from EVOO [13] phenols from lichens [11], and flavonoids [1,66]. LAEE-extracted *Cytisus scoparius* [1] were equivalent to methanolic extracts prepared under the same conditions. To our knowledge, there have been no reports of the use of LAEE in phenolic extraction from EVOOs.

#### 3.1.4. Interaction Effects

**Figure 2:**
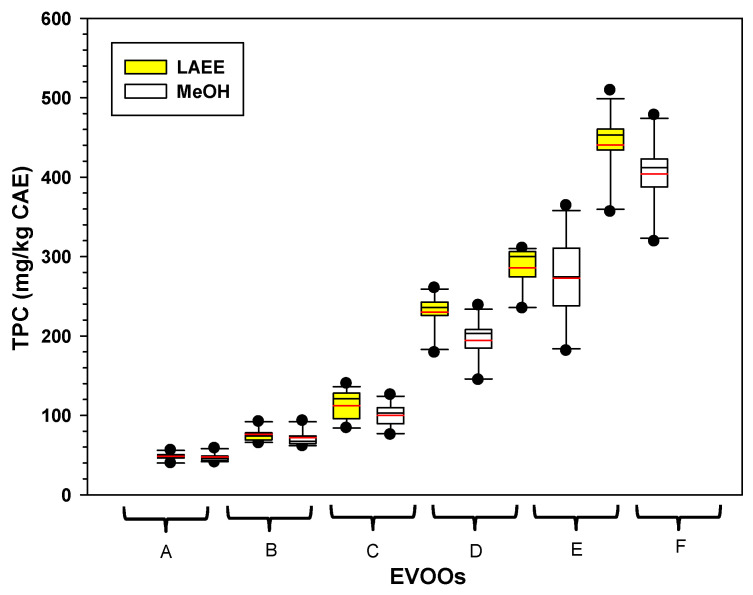
Box plot of the interaction effects and means for the variable TPC (phenolic compounds, mg CAE/kg) obtained from the extraction of six EVOOs using LAEE and MeOH at 1:4 to 5:0 OSWR (organic-solvent-to-water ratios, *v:v*). The red line within boxes represents mean values. A-F: represent EVOO samples.

Figure 2 shows the TPC of the interaction of EVOOs with organic solvents LAEE and MeOH at 1:4 to 5:0 OSWR (*v:v*). EVOOs with low phenolic content (A, B, and C, Figure 1A and Figure 2) had a low TPC when extracted with MeOH or LAEE.

Supplementary Figure S1 demonstrates that the interaction of EVOOs with OSWR rose linearly with the addition of water, and that the slope of this increase became gradually bigger as the TPC of EVOOs increased (see Supplementary Table S2 and Figure S2). There was a substantial linear association (r^2^ = 0.9963, *p* < 0.05) between MeOH and LAEE in relation to TPC, particularly at 4:1 (*v:v*) OSWR (see Supplementary Figure S3 for a detailed correlation plot).

### 3.2. RSA Antioxidant Activity

In this study, the EVOO factor and OSWR used had a large and significant impact (η^2^ₚ = 1.00) on the variable %RSA (displayed in Supplementary Table S1). However, the organic solvent effect was reduced and non-significant (η^2^ₚ = 0.65, <5%, *p* > 0.05), and its interaction with the organic solvent type and the OSWR created moderate effects.

#### 3.2.1. Effects of EVOOS

**Figure 3:**
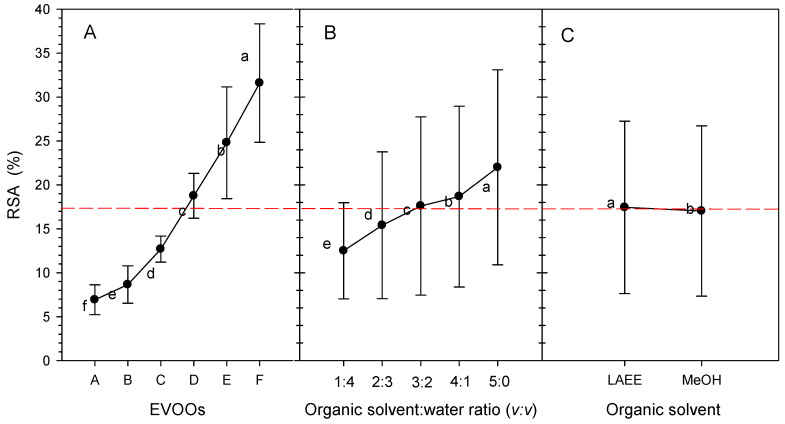
Main effects and means and standard deviations for variable radical scavenging activity (%RSA, DPPH assay) obtained from the extraction of (**A**) six EVOOs using (**B**) 1:4 to 5:0 organic-solvent-to-water ratios (*v:v*), with the organic solvents being (**C**) LAEE and MeOH. The dashed line represents the mean value of all factors. Different letters at each point represent statistically significant differences according to Tukey’s test.

The reported radical scavenging values, all produced with same DPPH method and expressed as %RSA, show a wide range from very low (5%) to very high, 98%. The reported ranges are 41.74–63.5% [67], 26.0–42.4% [68], 20–36% [69], 38.54–88.40% [70], 15–45% [71], 37.23–78.56% [46], 27.59–94.36 [72], 83–95% [73], 50–98% [74], 5–20% [75], 8.3–37.6% [76], 48–61% [77], and 14–88% [78]. The wide range of olive oils across countries, varieties, cultures, and processing methods reflects differences in the composition of their phenolic constituents as extracted with various organic solvent combinations [19,79,80]. However, it should not be overlooked that the lack of expression using a reference compound does not permit direct comparison due to differences in the adopted protocols. Apart from this, Zullo, and Ciafardini [79] reported that individual phenolics have varying %RSA values. Generally, it is estimated that 20% RSA might be considered the average for EVOOs. In this investigation, the average RSA of EVOOs was 17.5%, with a range of 5–40% (Figure 3).

To further understand the relationship between oil antioxidant activity and phenolic compound content, all the produced extracts were employed in an analysis of the correlation between TPC and %RSA. Supplementary Figure S4 reveals a strong linear association (r^2^ = 0.9192, *p* < 0.05) between TPC and %RSA, demonstrating that EVOOs with higher TPC have higher antioxidant capacity. Several researchers in different experimental setups have also reported a high association between TPC and %RSA. Minioti and Georgiou [59] reported r^2^ = 0.89, but Fanali et al. [81] reported r^2^ = 0.893, and Wani et al. [51] also found strong correlations between phenolic compounds and antioxidant properties (r^2^ > 0.8). Ballus et al. [82], r^2^ = 0.79; Samaniego Sánchez et al. [83], 0.79; and Negro et al. [84], r^2^ = 0.77, found less strong associations (r^2^ = 0.77–0.79). However, Rumpf et al. [85] reported modest correlations (r^2^ = 0.440).

Phenolic compounds in EVOOs have antioxidant properties because they can remove peroxyl and alkoxy radicals and chelate transition metal ions in trace amounts [17]. In terms of individual phenolic compounds, hydroxytyrosol has been shown to have stronger antiradical activity than oleuropein and caffeic acid [79]. Also, Lammi et al. [86] revealed that hydroxytyrosol and oleuropein appear to be better antioxidants than tyrosol due to the inclusion of an ortho-diphenolic group in their structure [19]. This is further supported by theoretical findings in a variety of olive phenols on the basis of -O-H bond dissociation enthalpy values [87].

#### 3.2.2. Effects of the Organic-Solvent-to-Water Ratio

Before %RSA measurement, EVOOs were extracted at OSWR ranging from 1:4 to 5:0 (*v:v*) using either LAEE or MeOH. The average RSA of EVOOs was 17.5%, but the range of EVOOs RSA extracted with at 1:4 to 5:0 OSWR was 12–21% (Figure 3B).

The average %RSA of the EVOOs in this study varied significantly according to the OSWR used (Figure 3B). The less water used to dilute the organic solvents, the greater the average %RSA; 3:2 OSWR (*v:v*) for extraction produced an average RSA of 18%, whereas 4:1 OSWR (*v:v*) was 19%. However, extraction with pure organic solvents (OSWR 5:0, *v:v*) resulted in the highest average RSA in extracts of 21%. Because of the many OSWRs utilized for extraction (e.g., MeOH/water ratios of 4:1, 3;2, and 1:1) as stated in Section 3.2.2, it is nearly impossible to compare these results to those reported in the literature.

#### 3.2.3. Effect of Organic Solvent

EVOOs extracted with MeOH or LAEE had an average RSA value of 17.5%, which did not differ significantly (<5%, *p >* 0.05), despite minor changes (Figure 3C).

#### 3.2.4. Interaction Effects

Supplementary Figure S5 shows the interaction of EVOOs with OSWR for MeOH and LAEE extraction solvents. A linear increase in %RSA was seen with the addition of water; additionally, the slope of this increase became progressively bigger as the %RSA of EVOOs increased (see Supplementary Table S2 and Figure S6). There was a significant linear association (r^2^ = 0.9963, *p* < 0.05) between MeOH and LAEE in relation to %RSA, particularly for 4:1 OSWR (see Supplementary Figure S7 for a detailed correlation plot).

**Figure 4:**
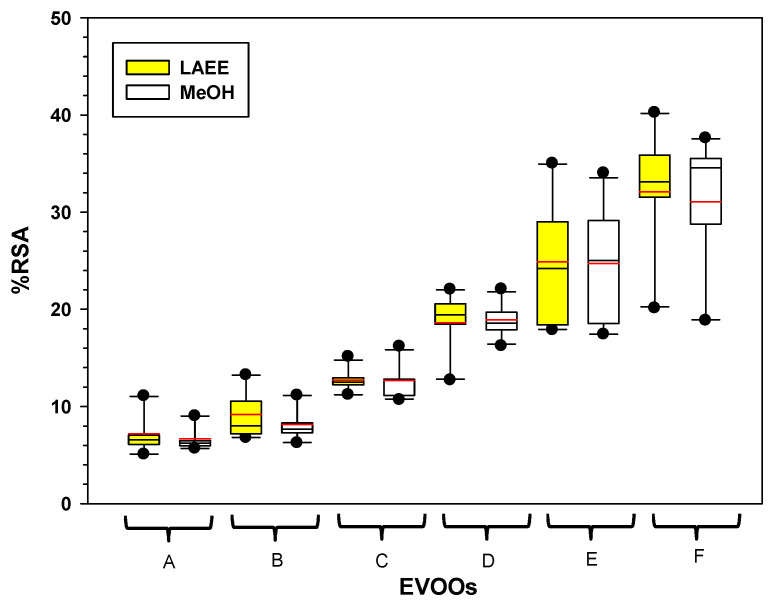
Box plot of the interaction effects and means for the variable %RSA (DPPH assay) obtained from the extraction of six EVOOs using LAEE and MeOH at 1:4 to 5:0 organic-solvent-to-water ratios (*v:v*). The red line within boxes represents mean values. A–F: represent EVOO samples.

Figure 4 shows the %RSA of the interaction between EVOOs, LAEE, and MeOH at 1:4 to 5:0 (*v:v*) OSWR. Low-phenolic EVOOs (A, B, and C) had low %RSA when extracted with either MeOH or LAEE, but high-phenolic EVOOs (D, E, and F) had higher %RSA when extracted with either LAEE or MeOH.

### 3.3. Phenolic Profiles

The extraction solution containing MeOH or LAEE:H_2_O (4:1, *v*/*v*) was selected to extract phenolic compounds from EVOOs according to the IOC method [35]. The recovered aqueous MeOH or LAEE phase was evaporated or freeze dried, respectively, before the phenolic compounds were dissolved in MeOH:H_2_O (1:1, *v:v*) and injected for HPLC profile analysis. LAEE is less volatile than MeOH; hence, freeze drying is required to remove the solvent prior to HPLC analysis. It should be emphasized that LAEE recovery is possible via distillation or membrane filtration [3], but these methods can be costly and risk phenolic degradation. Freeze-drying was used here to preserve analytes, but future industrial-scale recovery will require more efficient and gentle methods. While analytical results are promising, industrial use of LAEE requires overcoming challenges in solvent recovery and phenolic stability. Developing cost-effective and energy-efficient recovery methods is the key for scaling up.

An external calibration standard solution was made with methanol/water (4:1, *v*/*v*) and syringic acid (c = 0.015 mg/mL). Using syringic acid as the internal standard, the average recovery for MeOH and LAEE was 107.78 and 108.22, respectively, with RSD (%) of 1.11 and 1.75.

The validation of the proposed analytical method for the determination of individual phenols in EVOOS followed the IOC [36]. The concentration range, linearity, repeatability, limit of detection (LOD), and limit of quantitation (LOQ) were investigated. Table 1 provides the validation parameters that were tested. Depending on the analytes, the method’s linearity was investigated in various ranges. The correlation coefficient of determination (r^2^) for calibration curves was found to be ≥0.9961, showing a high linear response for all detected compounds. The lowest concentration of analytes that can be detected but not quantified is known as the LOD, whereas the lowest concentration of analytes that can be detected and precisely quantified is known as the LOQ. The findings showed that the LOQs for oleuropein and vanillin were 107.512 mg/kg and 0.056 mg/kg, respectively, and that the LODs for both ranged from 0.018 and 35.479 mg/kg. The percent relative standard deviation (%RSD) for replicates (n = 3) for all analytes was calculated to assess method precision; it was less than 0.197 (for oleacin), showing high repeatability.

**Figure 5:**
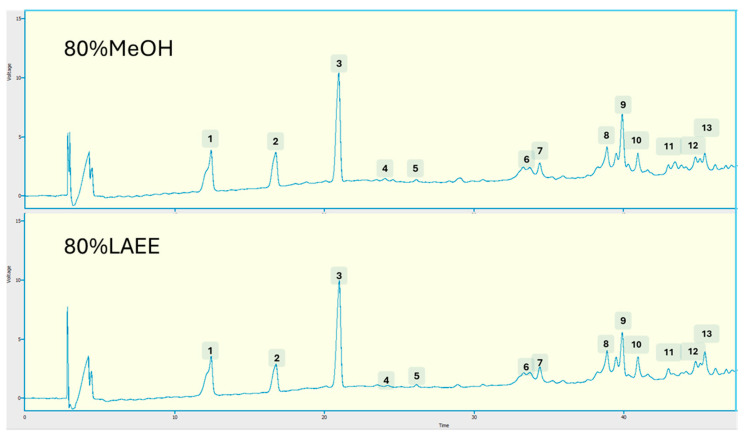
HPLC-DAD chromatogram of phenolic compounds extracted with 80% MeOH or LAEE/H_2_O (*v*/*v*) of one of the analyzed EVOO samples. 1: Hydroxytyrosol; 2: Tyrosol 3: Syringic acid (internal standard); 4: Vanillic acid; 5: p-coumaric acid; 6: Oleacein; 7: Oleuropein; 8: Oleocanthal; 9: Unknown; 10: Luteolin; 11: Unknown; 12: Unknown; 13: Apigenin.

The phenolic chemical profiles of the EVOOs differed only quantitatively; each EVOO had a similar profile for both the organic solvents, MeOH and LAEE, used (see Supplementary Figure S8 for chromatograms obtained from the analysis of EVOOs and organic solvents). Figure 5 depicts the individual phenolic compounds detected by comparing retention periods with those of the standards. As can be observed, the EVOO extracts with 4:1 of either MeOH or LAEE OSWR (*v:v*) contained secoiridoids (6–8 of the chromatogram), phenolic alcohols (compounds 1, 2), flavonoids (10, 13), and phenolic acids (4, 5). Three unknown compounds were also found in the extracts (9, 11, and 12).

TPC in EVOOs is known to be dominated by secoiridoids (oleuropein, oleacin, and oleocanthal), simple phenols (hydroxytyrosol, tyrosol), and flavonoids (luteolin and apigenin) [22,25,27].

**Figure 6:**
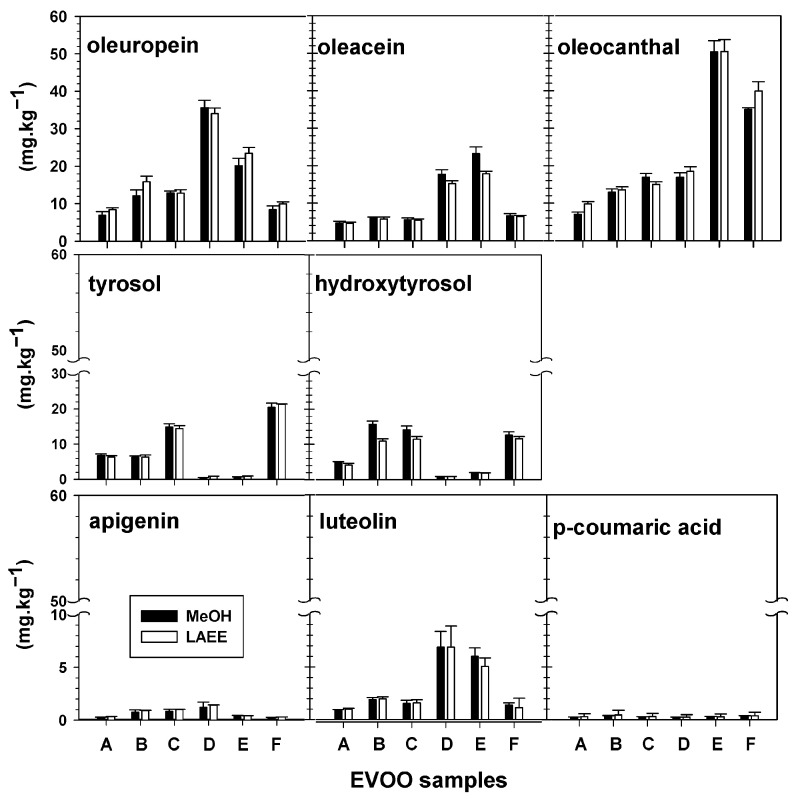
Trend of the major polyphenols (average plus SD) in six EVOOs extracted with 4:1 MeOH and LAEE OSWR. A–F: represent EVOO samples.

The results of phenolic profiles confirmed the comparative efficiency of LAEE to MeOH for recovering the range of phenolic compounds from EVOOs. Figure 6 shows the content of individual phenolics extracted by 4:1 OSWR of MeOH and LAEE. In all EVOOs, the secoiridoid class dominated, accounting for 70.3 and 72.3%, respectively; average oleuropein derivatives ranged between 16.0 and 17.4 mg/kg, respectively; and oleocanthal ranged between 23.2 and 24.5 mg/kg, while oleacin ranged between 10.6 and 95.2 mg/kg. Previous reports confirm the dominance of EVOO secoiridoids among phenolic compounds [88,89,90]. The presence of secoiridoids in olive oil contributes to its quality because of their antioxidative effects [91,92].

The content of the two simple phenolics, hydroxytyrosol and tyrosol, in EVOOs was 11.6 or 11.8 mg/kg for 4:1 OSWR MeOH and 11.71 or 9.6 mg/kg for 4:1 OSWR LAEE. Results for hydroxytyrosol in EVOOs A-C ranged from 6.7 to 14.4 mg/kg, whereas samples D and E ranged from 0.4 to 0.9 mg/kg for either MeOH or LAEE; these values were consistent with those reported in the literature. However, sample E had a significant hydroxy-tyrosol level of 21.3 mg/L. Tyrosol levels in EVOOs A–C or D and E followed a similar pattern, with concentrations of 11.6 or 12.6 mg/L in F (Figure 6). These contents were all within the reported range [93,94].

Simple phenols (p-coumaric and vanillic acids) and cinnamic were found in 0.1–0.6% (*w*/*w*) of the examined TPC. Phenolic acids are reported to have concentrations ranging from 0.01 to 1.7 mg/kg [44].

Apigenin and luteolin, the two most common flavonoids found in EVOO extracts, varied from 0.1 to 4.3% for both 80% MeOH and LAEE. Several publications have reported that flavonoids like luteolin and apigenin are phenolic components of EVOO [95].

Similar phenolic levels with MeOH and LAEE indicate broadly comparable solubility, despite different solvent properties. Although exact solubility data are lacking, results support ethyl lactate as a viable alternative.

A TPC of at least 250 mg/kg is often required for the European Union’s health claim [96], which links phenols to reduced oxidative stress in humans. Many commercial EVOOs contain 100–250 mg/kg, and this was the case in two (out of six) EVOOs, E and F. In general, the percentage of oils with phenolics greater than 250 mg GAE/kg (providing a valid health claim) ranges from 3 [44] to 30% [97].

The comparable extraction efficiency of LAEE relative to MeOH arises from its favorable physicochemical characteristics. LAEE’s hydrogen-bonding capacity, amphiphilic nature, and intermediate dielectric constant enable it to effectively solvate phenolic compounds within the predominantly non-polar olive oil matrix. Theoretical LogP values reported by Nenadis et al. [98] indicate moderate lipophilicity for tyrosol and related compounds such as oleocanthal (LogP 1.04–1.23), and greater polarity for hydroxytyrosol and related compounds (LogP 0.65–0.94). Additionally, the presence of phenolic hydroxyl and aldehyde functional groups in these analytes facilitates hydrogen bonding with LAEE, contributing to efficient extraction. These factors collectively explain the broadly comparable extraction performance of LAEE and MeOH observed in this study.

While analytical results are promising, industrial use of ethyl lactate requires overcoming challenges in solvent recovery and phenolic stability. Developing cost-effective and energy-efficient recovery methods is key for scaling up.

#### Principal Component Analysis

Principal components analysis (PCA) was used to analyze data on the concentration of individual phenolics, as well as TPC and %RSA of EVOOs after extraction with 4:1 OSWR of MeOH or LAEE (*v*:*v*). Figure 6 shows the score plot of PCA and the separation of organic solvents and EVOOs in this study. Based on the high eigenvalues (6.12 and 3.25), two principal components were considered: PC1 contributed 51%, and PC2 contributed 27.2% of the overall variability of the data. While there was no organic solvent separation, all data for EVOOs were separated into three groups: “A, B, C”, “D, E”, and “F”.

The “A, B, C” group has PC1 values that are negative, whereas the "D, E," and "F" groups have positive values. This is due to the “A, B, C” group having a lower phenolic/%RSA concentration than the other groups. Furthermore, the “A, B, C,” and “F” groups showed higher PC2 values than the “F” group, indicating differences in their phenolic profiles (EVOOs D and E had low levels of hydroxytyrosol and tyrosol).

### 3.4. Acid Hydrolysis

Complete recovery of phenols from the olive oil matrix poses a difficult task, as they represent a varied range of molecules with considerable structural diversity and physicochemical behavior [14].

**Figure 7:**
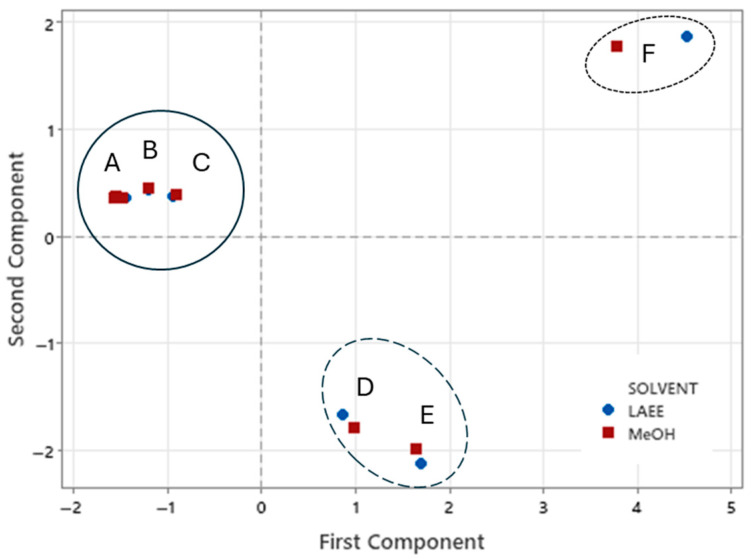
Principal component analysis (PCA): score of PC1 and PC2 after analysis of the effects of extraction of six EVOOs with 4:1 organic solvent/water (MeOH or LAEE) ratios (*v:v*) on TPC, RSA, and individual phenolics obtained from the HPLC profile. Key of abbreviations (attributes): A–F represents EVOOs samples. Key of symbols: EVOOs of low TPC/RSA are shown by an ellipse with a solid line, whereas intemedeate and high TPC/RSA are indicated by dashed and dotted lines, respectively.

**Figure 8:**
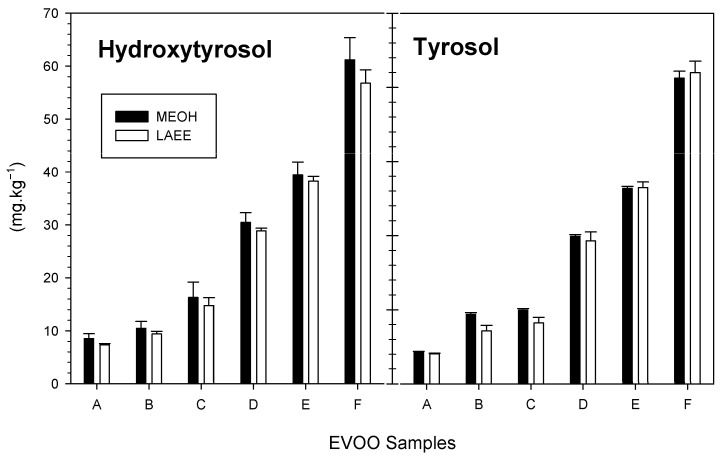
Total hydroxytyrosol and tyrosol content (average plus SD) of six EVOOs (A–F) following extraction with 4:1 MeOH or LAEE OSWR (*v:v*) followed by acid hydrolysis.

Considering the complexity of the chromatographic profile of the EVOOs (Figure 7) and to better evaluate the performance of the two organic solvents, acid hydrolysis of the extracts from the EVOOs extracted with 4:1 MeOH or LAEE OSWR (*v:v*) was applied, according to the Olive Council method [36]. This approach is well established for simplifying the phenolic profile and revealing more clearly the presence of bound forms of hydroxytyrosol and tyrosol, aligning with the mandates of the European Commission [96,99,100]. The quantitative results are presented in Figure 8. The acid hydrolysis procedure was applied as described by Mulinacci et al. [42] and further validated by Tsimidou et al. [101]. As recommended by [100], chromatograms of the hydrolyzed samples were inspected (Figure S9), and no peaks related to degradation artifacts were observed.

Following hydrolysis, higher peaks of hydroxytyrosol and tyrosol were evidenced verifying the presence of corresponding bound forms. The MeOH extracts tended to produce higher yields than LAEE extracts (Figure 8), with concentrations ranging from 8 to 80 mg/kg oil, varying by EVOOs, extractants, and phenolic species. Other phenolic acids (e.g., *p*-coumaric, vanillic) were not detected in the hydrolyzed extracts (Supplementary Figure S9).

The amount of hydroxytyrosol or tyrosol recovered during acid hydrolysis increased by 1.2–4.5 mg/kg in EVOOs A-C (Figure 8) when compared to values obtained from profile analysis (Figure 6). EVOOs D and E with very low hydroxytyrosol or tyrosol content after acid hydrolysis contained 30.4 and 39.3 mg/kg, respectively, following extraction with 4:1 OSWR of MeOH or 28.8 and 38.2 mg/kg after extraction with 4:1 OSWR of LAEE. These findings indicate that esterified connections occurred with the majority of simple phenolic compounds, with the exception of hydroxytyrosol, which is expected to be easily released during the extraction process [102]. In the same context, EVOO sample E had the highest levels of hydroxytyrosol and tyrosol, which resulted from both the increased content revealed in profile analysis (Figure 6) and the hydrolysis of esterified complex phenols.

## 4. Conclusions

Six EVOOs were extracted for phenolic compounds using aqueous MeOH and LAEE at 1:4 to 5:0 (organic-solvent-to-water ratios, *v:v*). The total phenolic content and antioxidant activity of EVOO extracts were assessed. The concentrations of phenolic compounds recovered from EVOOs were comparable with either organic solvent (MeOH or LAEE). The TPC and percentage of the antioxidant radical scavenging activity of EVOOs increased in both organic solvents (MeOH and LAEE) as the amount of water in mixtures decreased (5:0 > 4:1 > 3:2 > 2:4 > 1:4 organic solvent/water, *v:v*).

The phenolic profile of 4:1 OSWE (*v:v*) was determined for both organic solvents. A single extraction is recommended for a quick but valid assessment; however, for a more thorough recovery, a second or even triple extraction of the same EVOO sample may be required. It is concluded that LAEE at 4:1 OSWE (*v:v*) can be efficiently used in the extraction and recovery of the main bioactive compounds from olive oil, including phenolic alcohols and secoiridoids. Although LAEE extraction requires freeze-drying to remove the solvent—introducing additional operational steps compared to MeOH extraction—the method remains effective and environmentally favorable as a green alternative. Nevertheless, MeOH is less expensive. LAEE is advised for green extraction of olive oil’s phenolic fraction to analyze or determine total phenolics and radical scavenging activity. The restriction of using LAEE extraction for phenolic profile HPLC analysis is that it requires freeze drying.

## Figures and Tables

**Table 1:** HPLC-DAD method validation parameters for quantification of olive phenolic compounds.

	Concentration Range (mg/kg)	Determination Coefficient (r^2^)	Repeatability of Retention Time (%RSD)	LOD	LOQ
Luteolin	0.5–10	0.9971	0.100	0.709	2.149
Vanillic acid	0.05–10	1	0.129	0.037	0.111
Hydroxytyrosol	7.5–100	0.9997	0.183	2.154	6.526
Tyrosol	7.5–100	1	0.100	0.185	0.560
Oleacin	7.5–100	0.9961	0.197	8.422	25.520
Oleuropein	7.5–200	0.9981	0.089	35.479	107.512
Oleocanthal	7.5–100	0.9997	0.056	2.497	7.567
Cinnamic acid	0.1–1	0.9987	0.111	0.048	0.144
Vanillin	0.1–1	0.9982	0.064	0.018	0.056
*p*-Coumaric acid	0.2–10	0.9999	0.074	0.103	0.312
Apigenin	0.1–5	1	0.027	0.028	0.084
Ferulic acid	0.1–10	0.9996	0.158	0.019	0.058

RSD: relative standard deviation; LOD: limit of detection (mg/kg of EVOO); LOQ: limit of quantification (mg/kg of EVOO).

## Data Availability

The data presented in this study are available on request from the corresponding author due to privacy restrictions.

## Supplementary Materials

The following supporting information can be downloaded at https://www.mdpi.com/article/10.3390/foods14223822/s1, Figure S1: TPC of six EVOOs (A–E) extracted by 1:4 to 5:0 organic-solvent-to-water ratios (*v*:*v*), of MeOH (A) or LAEE (B). Solid lines represent linear regression and dashed ones 95% confidence intervals; Figure S2. Linear regression of the coefficients (slopes) obtained from Table S2. Solid lines are linear regression lines, dashed 95% and dotted 99% intervals. Linear regressions of the slope values obtained from plots of TPC-MeOH vs. TPC-LAEE (1:4 to 5:0 organic-solvent-to-water ratios *v*:*v*) from six EVOOs (A–E). Solid lines represent the fitted regression models, while dashed and dotted lines indicate the 95% and 99% confidence intervals, respectively; Figure S3. TPC of EVOO samples extracted with 4:1 organic-solvent-to-water ratios (*v*:*v*), of MeOH or LAEE. The enclosed figure within the graph (A1) depicts the linear relationship between TPC values of 4:1 MeOH vs. 4:1 LAEE extracts; Figure S4. Linear regression of TPC vs. %RSA (DPPH) values of six EVOOs extracted by 1:4 to 5:0 organic-solvent-to-water ratios (*v*:*v*) of MeOH or LAEE. Solid red line is linear regression line, dashed lines 95% intervals and dotted lines prediction intervals; Figure S5. % RSA (DPPH) values of six EVOOs(A–E) extracted with 1:4 to 5:0 organic-solvent-to-water ratios (*v*:*v*) of MeOH (A) or LAEE (B). Solid lines represent linear regression and dashed ones 95% confidence intervals; Figure S6. Linear regressions of the slope values obtained from plots of %RSA values (DPPH) against the organic-solvent-to-water ratios (*v*:*v*) of MeOH or LAEE used for extraction of six EVOOs (A–E). Solid lines represent the fitted regression models, while dashed and dotted lines indicate the 95% and 99% confidence intervals, respectively; Figure S7. %RSA (DPPH) of EVOOs extracted with 4:1 organic-solvent-to-water ratios (*v*:*v*) of MeOH or LAEE. The enclosed figure (A1) within the graph depicts the linear relationship between %RSA values obtained for MeOH extracts vs. LAEE extracts; Figure S8. HPLC phenolic profile of six EVOOs (A–E) following extraction with 4:1 aqueous MeOH (A) and 4:1 aqueous LAEE (B); Figure S9. HPLC phenolic profile of six EVOOs (A–E) following extraction with 4:1 aqueous MeOH (A) and 4:1 aqueous LAEE (B) after acid hydrolysis; Table S1. Partial eta-squared values for the variables phenolic compounds (TPCs, mg CAE/kg), and %RSA of extra virgin olive oil samples extracted with 1:4 to 5:0 organic-solvent-to-water ratios (*v*:*v*), the factor solvent being LAEE or MeOH; Table S2. Linear regression coefficients (+STD) for TPC and %RSA (DPPH) of six EVOOs (A–F) extracted with 1:4 to 5:0 organic-solvent-to-water ratios (*v*:*v*), of MeOH or LAEE; Table S3. Linear regression equations and coefficients for analyte standards run for HPLC profiling. Table S4. Average concentrations (mg/Kg) of the phenolic compounds investigated in the EVOOs following HPLC-DAD profile analysis.
