# Supplementary material for: Assessment of the Functional Quality of Extra Virgin Olive Oil: Green Extraction of Phenolic Compounds Using Ethyl Lactate
Source: Foods. 2025 Nov 7;14(22):3822. doi: 10.3390/foods14223822 (PMC12650932; doi:10.3390/foods14223822)
Supplement: Supplementary file 1 [file foods-14-03822-s001.zip › foods-3953843-supplementary.pdf]

foods

MDPI

Supplementary Material

Assessment of the Functional Quality of Extra Virgin Olive Oil: Green Extraction of Phenolic Compounds Using Ethyl Lactate

Chrysostomos Tsitsipas [1], Athanasios Gerasopoulos [2], Nikolaos Nenadis [2] and Dimitrios Gerasopoulos [1,*]

[1] Laboratory of Food Processing and Engineering, Department of Food Science and Technology, Faculty of Agriculture, Aristotle University of Thessaloniki, 54124 Thessaloniki, Greece; ctsitsip@agro.auth.gr
[2] Laboratory of Food Chemistry and Technology, School of Chemistry, Aristotle University of Thessaloniki, 54124 Thessaloniki, Greece; ageraso@chem.auth.gr (A.G.); niknen@chem.auth.gr (N.N.)
* Correspondence: dgerasop@agro.auth.gr; Tel.: +30-2310-991643

2.7. Statistical analysis

The normality of dependent variables was tested using the Anderson-Darling test and partial eta squared ($\eta^2{}_p$) was calculated to evaluate the effects of each factor ($\eta^2{}_p$= SSeffect/(SSeffect + SSerror), where SS = sum of squares).

3.1. Analysis of Variance-partial eta-squared

The study's data (dependent variables) were TPC and antioxidant activity. Partial eta-squared ($\eta^2{}_p$) is an effect size measure, that quantifies the proportion of variance in a dependent variable explained by a specific independent variable or factor, after controlling for the variance explained by other factors in the model.

Table S1. Partial eta-squared values for the variables phenolic compounds (TPCs, mg CAE/kg), and %RSA of extra virgin olive oil samples extracted with 1:4 to 5:0 organic solvent to water ratios (v:v), the factor solvent being LAEE or MeOH.

	DF	TPC	%RSA
		PARTIAL ETA SQR ($\eta^2{}_p$)	
EVOO (A)	2	1,00 ***	1,00 ***
SOLVENT:WATER (B)	1	0,98 ***	1,00 ***
SOLVENT (C)	1	0,85 ***	0,65 ns
A*B	4	0,95 ***	1,00 ***
A*C	2	0,80 ***	0,73 ***
B*C	2	0,54 ***	0,73 ***
A*B*C	8	0,76 ***	0,93 ***

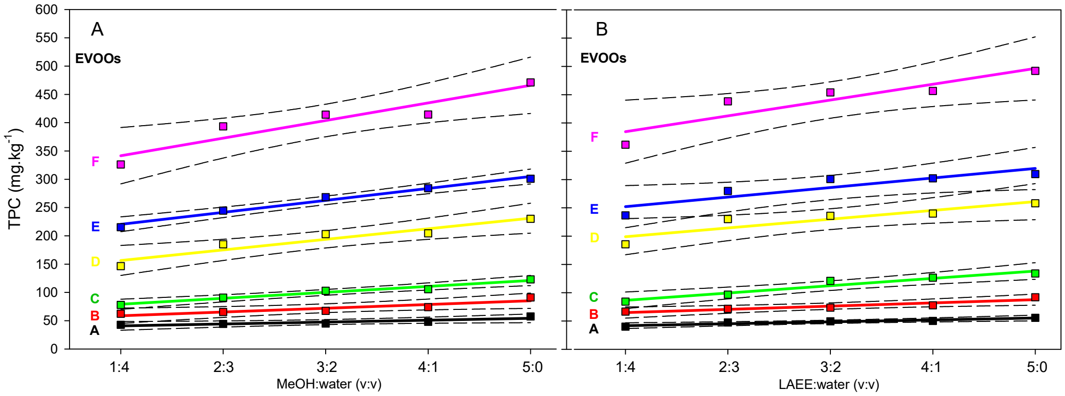

Figure S1. TPC of six EVOOs (A-E) extracted by 1:4 to 5:0 organic solvent to water ratios (v:v), of MeOH (A) or LAEE (B). Solid lines represent linear regression and dashed ones 95% confidence intervals.

Table S2. Linear regression coefficients (±STD) for TPC and %RSA (DPPH) of six EVOOs (A-F) extracted with 1:4 to 5:0 organic solvent to water ratios (v:v), of MeOH or LAEE.

EVOOs		Linear regrassion f(x)=yo+a*x coeficients					
		y_o	a	R^2	y_o	a	R^2
		MeOH			LAFE		
A	TPC	37.50±3.27 **	0.16±0.04 *	0.7927	38.33±2.16 ***	0.16±0.03 *	0.8998
B		52.13±5.75 **	0.33±0.08 *	0.8295	59.20±4.10 ***	0.28±0.06 *	0.8720
C		68.57±3.79 ***	0.52±0.05 **	0.9655	73.62±6.27 ***	0.64±0.09 **	0.9396
D		137.80±11.2 **	0.93±0.17 *	0.9095	183.48±13.56 ***	0.77±0.20 *	0.8269
E		199.28±5.50 ***	1.05±0.08 **	0.9812	235.25±15.84 ***	0.84±0.23 *	0.8059
F		310.5±21.21 ***	1.55±0.31 *	0.8874	356.69±23.72 ***	1.39±0.35 *	0.8357
\	%RSA	4.65±0.82 *	0.039±0.01 ns	0.7147	3.41±1.28 ns	0.06±0.01 *	0.7781
B		4.99±0.80 **	0.052±0.01 *	0.8626	4.59±0.93 *	0.07±0.01 *	0.9107
C		9.19±0.99 **	0.059±0.01 *	0.8413	10.43±0.64 ***	0.04±0.01 *	0.8591
D		15.70±0.43 ***	0.054±0.01 **	0.9585	12.60±1.60 **	0.10±0.02 *	0.8508
E		12.15±1.32 **	0.21±0.02 **	0.9735	11.62±1.93 **	0.22±0.02 **	0.9498
F		18.10±3.52 *	0.21±0.05 *	0.8487	20.24±4.14 *	0.19±0.06 ns	0.7623

Significant at *95%, **99%, ***99.9%

TPC readings from EVOOs extracted with 1:4 to 5:0 organic solvent to water ratios (v:v), of MeOH or LAEE were also analyzed using linear regression (Figure S1). Table S2 includes the equation coefficients a (slope) and yo (constant), as well as R^2 (coefficient). The slope (a) for TPC following extraction with MeOH of low in TPC EVOOs (A and B) had range values of 0.16-0.33, while in intermediate it increased to range values of 0.52-0.93 in (C and D) and in high (E and F) in TPC samples to values of 1.05-1.55; TPC extraction using LAEE produced similar though lower slope values.

In a further analysis it was demonstrated a significantly strong linear regression (P<0.000, R^2=0.9468) between the slope values of the two extraction methods (MeOH and LAEE) for TPC (Figure S2).

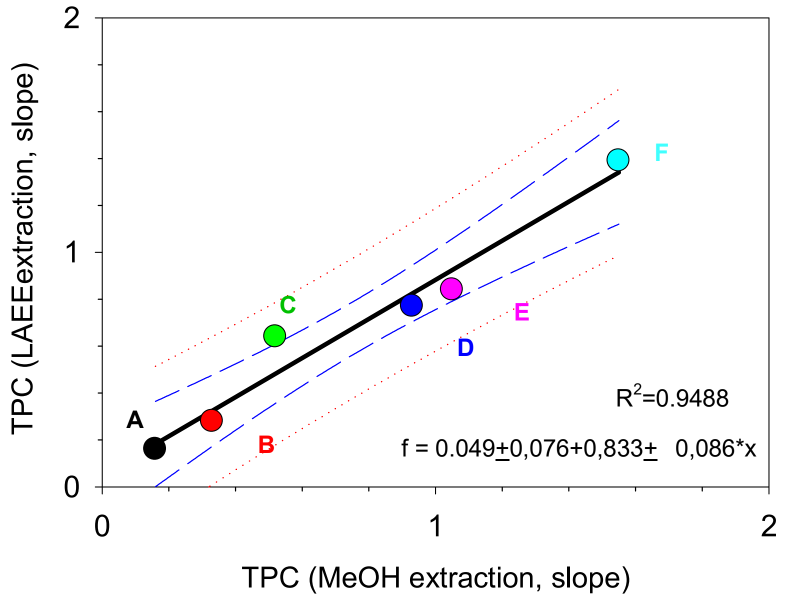

Figure S2. Linear regression of the coefficients (slopes) obtained from Table S2. Solid lines are linear regression lines, dashed 95% and dotted 99% intervals. Linear regressions of the slope values obtained from plots of TPC-MeOH vs TPC-LAEE (1:4 to 5:0 organic solvent to water ratios *v:v*) from six EVOOs (A–E). Solid lines represent the fitted regression models, while dashed and dotted lines indicate the 95% and 99% confidence intervals, respectively.

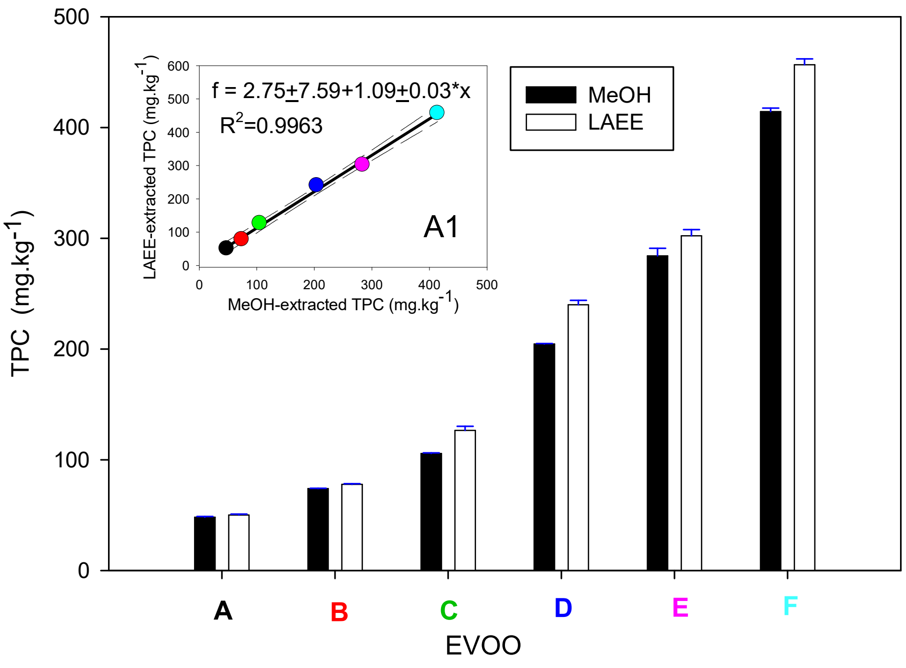

Figure S3. TPC of EVOO samples extracted with 4:1 organic solvent to water ratios (v:v), of MeOH or LAEE. The enclosed figure within the graph (A1) depicts the linear relationship between TPC values of 4:1 MeOH vs 4:1 LAEE extracts.

Figure S3 shows the comparative values for 4:1 organic solvent to water ratios (v:v) of MeOH or LAEE extracts for TPC; LAEE extracts outperformed MeOH for all EVOO samples in phenolic content; a strong linear correlation (*p*<0.0001, R^2= 0.99) between 4:1 organic solvent to water ratios (v:v) of MeOH and LAEE extracts was observed the phenolic content (Figure S3A1).

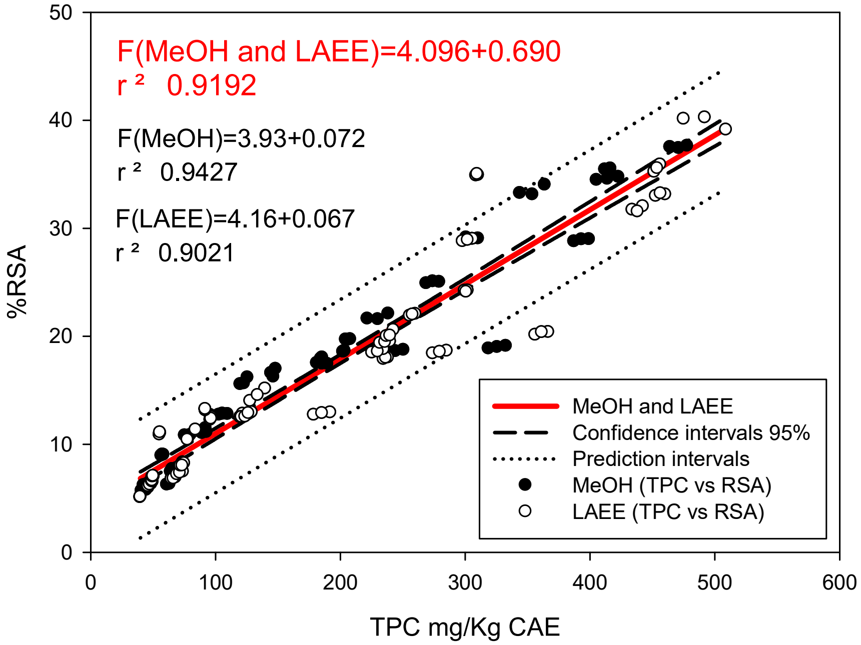

Figure S4. Linear regression of TPC vs %RSA (DPPH) values of six EVOOs extracted by 1:4 to 5:0 organic solvent to water ratios (*v:v*) of MeOH or LAEE. Solid red line is linear regression line, dashed lines 95% intervals and dotted lines prediction intervals.

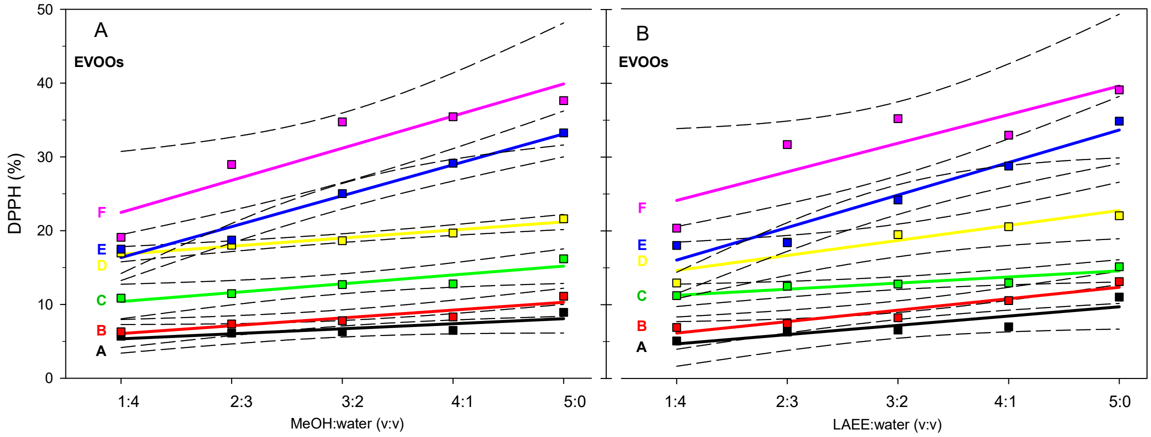

Figure S5. % RSA (DPPH) values of six EVOOs(A-E) extracted extracted with 1:4 to 5:0 organic solvent to water ratios (*v:v*) of MeOH (A) or LAEE (B). Solid lines represent linear regression and dashed ones 95% confidence intervals.

%RSA from EVOOs extracted with 1:4 to 5:0 organic solvent to water ratios (*v:v*) of MeOH or LAEE were also analyzed using linear regression. Table S2 includes the equation coefficients a (slope) and yo (constant), as well as R^2 regression coefficient. The slope (a) for %RSA following extraction with MeOH of low in TPC EVOO samples (A and B) had range values of 0.16-0.33, while in intermediate it increased to range values of 0.52-0.93 in (C and D) and in high (E and F) in TPC samples to values of 1.05-1.55; %RSA extraction using LAEE produced similar though lower slope values.

In further analysis (Figure S6) was demonstrated a linear regression of the correlation of slope values for %RSA antiradical activity was less strong (p<0.000, R^2=0.8999).

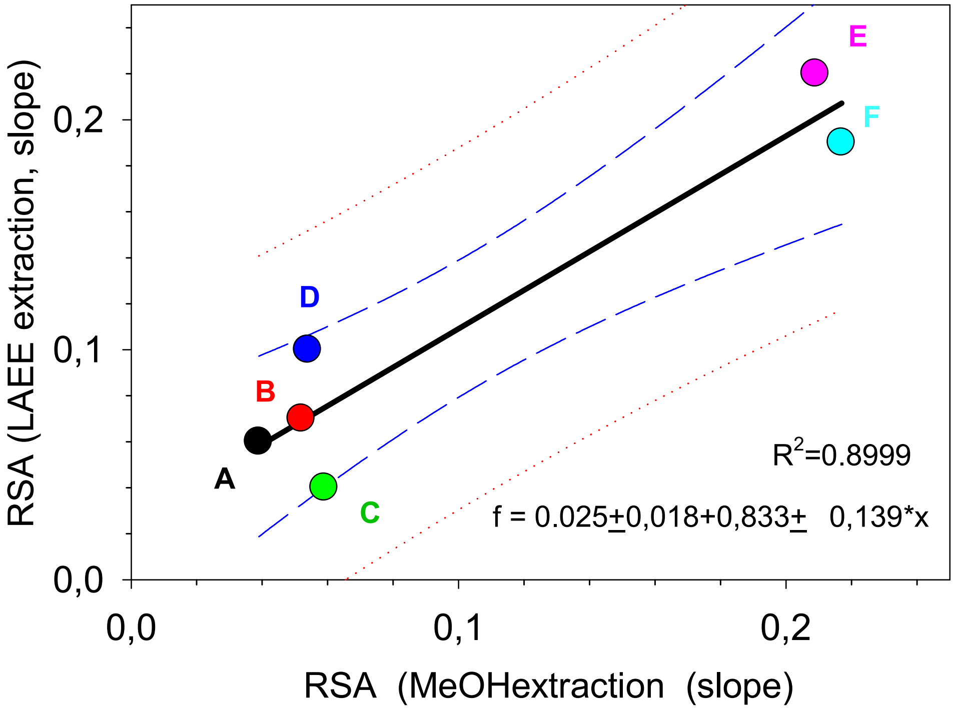

Figure S6. Linear regressions of the slope values obtained from plots of %RSA values (DPPH) against the organic solvent to water ratios (*v:v*) of MeOH or LAEE used for extraction of six EVOOs (A–E). Solid lines represent the fitted regression models, while dashed and dotted lines indicate the 95% and 99% confidence intervals, respectively.

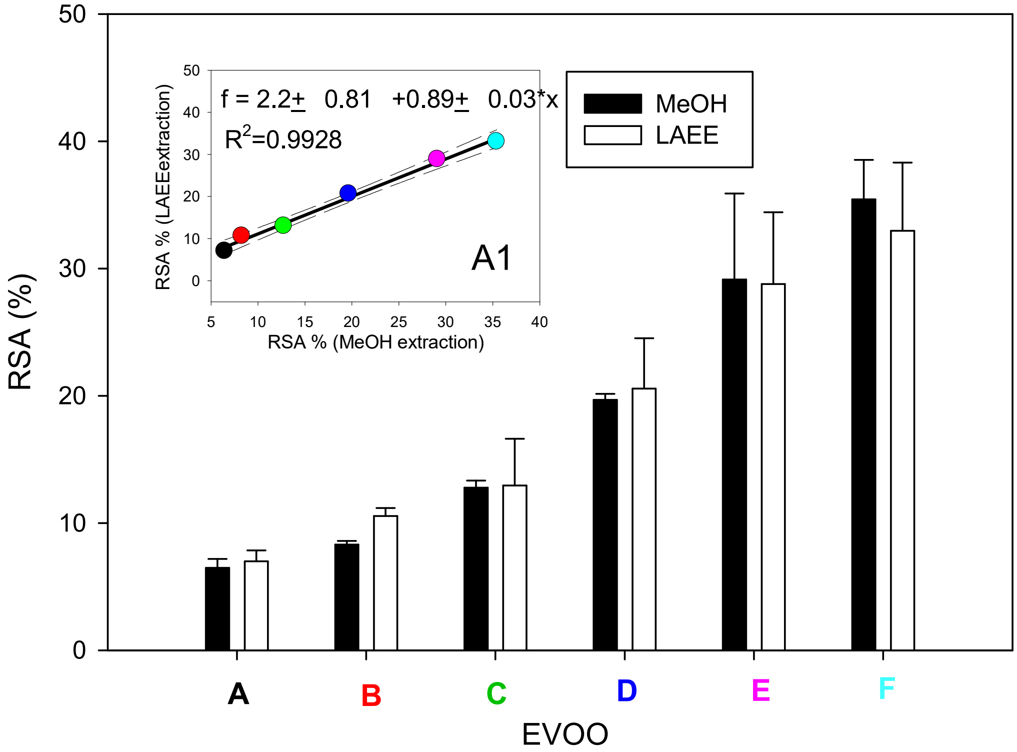

Figure S7. %RSA (DPPH)of EVOOs extracted with 4:1 organic solvent to water ratios (*v:v*) of MeOH or LAEE. The enclosed figure (A1) within the graph depicts the linear relationship between %RSA values obtained for MeOH extracts vs LAEE extracts.

The comparative values for 4:1 MeOH or LAEE aqueous extracts for %RSA antiradical activity is depicted in Figure S7; %RSA antiradical activity of LAEE extracts did not outperform MeOH for all EVOO samples as was the case for the phenolic content (Figure S3). However, a strong linear correlation (p<0.0001, R^2= 0.99) between 4:1 MeOH and4:1 LAEE aqueous extracts was observed in %RSA (Figure S7A1).

Table S3. Linear regression equations and coefficients for analyte standards run for HPLC profiling.

**Analyte standard**	**Equation**	**Determination coefficient (R^2)**
Luteolin	Y=33.806x-3.0505	0.9971
Vanillic acid	Y=43.844x-1.4888	0.9997
Hydroxytyrosol	Y=8.9274x-0.1109	0.9991
Tyrosol	Y=5.5832x-0.5567	1.000
Oleacein	Y=3.4194x-9.8961	0.9961
Oleuropein	Y=2.5075x-10.850	0.9981
Oleocanthal	Y=1.8415x-3.7209	0.9997
Cinnamic acid	Y=79.612x-3.3339	0.9987
Vanillin	Y=36.321x-0.5245	0.9982

p-coumaric acid	Y=37.016x-3.5824	0.9997
Apigenin	Y=32.173x-0.4239	1.000
Ferulic acid	Y=45.865x+0.1323	0.9996

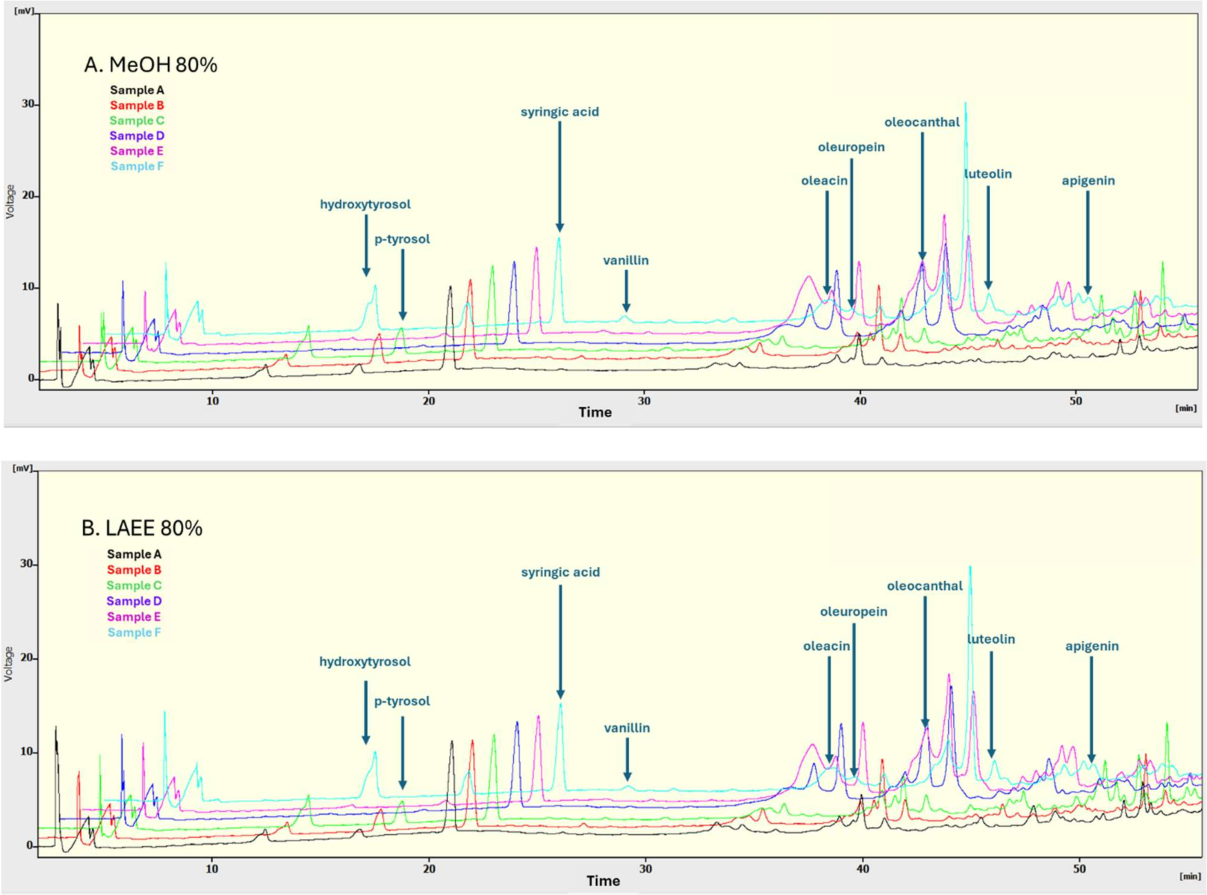

Figure S8. HPLC phenolic profile of six EVOOs (A-E) following extraction with 4:1 aqueous MeOH (A) and 4:1 aqueous LAEE (B).

Table S4. Average concentrations (mg/Kg) of the phenolic compounds investigated in the EVOOs following HPLC-DAD profile analysis.

EVOOs	Oleuropein	Oleacin	Oleocanthal	Hydroxytyrosol	Tyrosol	Apigenin	Lutein	Cinnamic acid	Coumaric acid	Vanilic acid
					MeOH					
A	6.91 ± 1.0	4.74 ± 0.4	7.01 ± 0.6	6.74 ± 0.5	4.79 ± 0.3	0.26 ± 0.0	0.85 ± 0.1	n.d.	0.25 ± 0.0	n.d.
B	12.12 ± 1.5	5.98 ± 0.3	12.93 ± 0.9	6.24 ± 0.4	15.71 ± 0.9	0.76 ± 0.2	1.90 ± 0.2	n.d.	0.38 ± 0.0	n.d.
C	12.90 ± 0.5	5.55 ± 0.5	16.92 ± 1.0	14.91 ± 0.9	14.12 ± 1.1	0.83 ± 0.2	1.54 ± 0.3	0.109 ± 0.0	0.27 ± 0.0	0.16 ± 0,8
D	35.63 ± 2.0	17.73 ± 1.2	16.91 ± 1.2	0.52 ± 0.0	0.85 ± 0.0	1.20 ± 0.5	6.87 ± 1.5	n.d.	0.22 ± 0.0	n.d.
E	20.13 ± 2.0	23.21 ± 1.8	50.36 ± 3.0	0.43 ± 0.3	1.69 ± 0.3	0.43 ± 0.0	5.99 ± 0.8	n.d.	0.30 ± 0.0	n.d.
F	8.40 ± 1.0	6.59 ± 0.6	35.00 ± 0.5	20.45 ± 1.2	12.63 ± 0.9	0.24 ± 0.0	1.40 ± 0.2	n.d.	0.37 ± 0.0	0.21 ± 0,6
					LAEE					
A	8.41 ± 0.5	4.58 ± 0.3	9.80 ± 0.6	6.30 ± 0.4	4.09 ± 0.5	0.33 ± 0.0	0.99 ± 0.1	n.d.	0.28 ± 0.3	n.d.
B	15.82 ± 1.5	5.79 ± 0.5	13.54 ± 0.8	6.33 ± 0.6	10.92 ± 0.7	0.91 ± 0.0	1.97 ± 0.2	n.d.	0.45 ± 0.4	n.d.
C	12.77 ± 1.0	5.40 ± 0.4	14.99 ± 0.7	14.44 ± 0.8	11.44 ± 0.8	1.00 ± 0.0	1.58 ± 0.3	0.00 ± 0.0	0.30 ± 0.3	n.d.
D	34.01 ± 1.5	15.22 ± 0.8	18.51 ± 1.2	0.91 ± 0.0	0.85 ± 0.1	1.40 ± 0.1	6.87 ± 2	n.d.	0.24 ± 0.2	n.d.
E	23.49 ± 1.5	17.81 ± 0.7	50.45 ± 3.2	0.91 ± 0.0	1.84 ± 0.1	0.40 ± 0.0	5.06 ± 0.8	n.d.	0.27 ± 0.3	n.d.
F	9.99 ± 0.5	6.42 ± 0.3	39.92 ± 2.5	21.31 ± 0.1	11.62 ± 0.6	0.30 ± 0.0	1.14 ± 0.9	n.d.	0.36 ± 0.4	n.d.

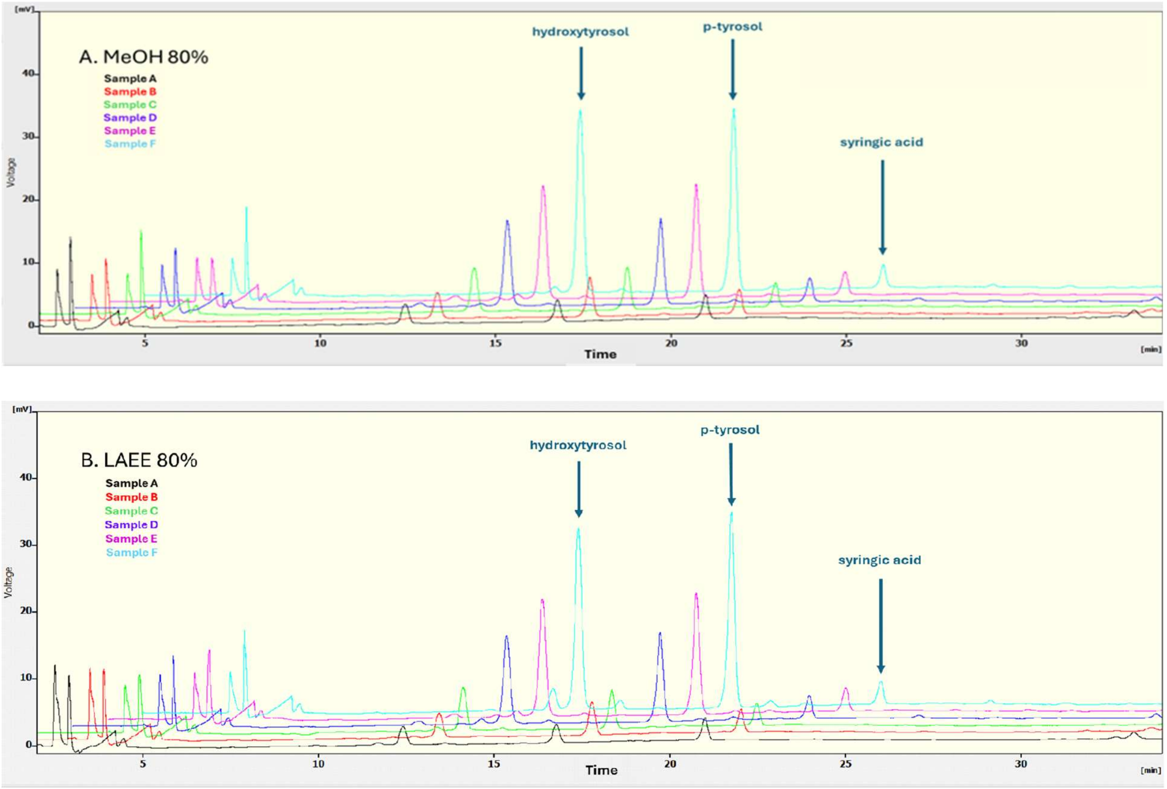

Figure S9. HPLC phenolic profile of six EVOOs (A-E) following extraction with 4:1 aqueous MeOH (A) and 4:1 aqueous LAEE (B) after acid hydrolysis.
